# Radiologist-Level Performance by Using Deep Learning for Segmentation of Breast Cancers on MRI Scans

**DOI:** 10.1148/ryai.200231

**Published:** 2021-12-15

**Authors:** Lukas Hirsch, Yu Huang, Shaojun Luo, Carolina Rossi Saccarelli, Roberto Lo Gullo, Isaac Daimiel Naranjo, Almir G. V. Bitencourt, Natsuko Onishi, Eun Sook Ko, Doris Leithner, Daly Avendano, Sarah Eskreis-Winkler, Mary Hughes, Danny F. Martinez, Katja Pinker, Krishna Juluru, Amin E. El-Rowmeim, Pierre Elnajjar, Elizabeth A. Morris, Hernan A. Makse, Lucas C. Parra, Elizabeth J. Sutton

**Affiliations:** From the Department of Biomedical Engineering (L.H., Y.H., L.C.P.) and the Benjamin Levich Institute and Department of Physics (S.L., H.A.M.), the City College of the City University of New York, 160 Convent Ave, New York, NY 10031; Department of Radiology, Memorial Sloan Kettering Cancer Center, New York, NY 10065 (Y.H., C.R.S., R.L.G., I.D.N., A.G.V.B., N.O., E.S.K., D.L., D.A., S.E.W., M.H., D.F.M., K.P., K.J., A.E.E., P.E., E.A.M., E.J.S.); Department of Imaging, A.C. Camargo Cancer Center, São Paulo, Brazil (A.G.V.B.); Department of Radiology, University of California, San Francisco, San Francisco, Calif (N.O.); Department of Radiology, Samsung Medical Center, Sungkyunkwan University School of Medicine, Seoul, Korea (E.S.K.); and Department of Breast Imaging, Breast Cancer Center TecSalud, ITESM Monterrey, Monterrey, Mexico (D.A.).

**Keywords:** MRI, Breast, Segmentation, Supervised Learning, Convolutional Neural Network (CNN), Deep Learning Algorithms, Machine Learning Algorithms

## Abstract

**Purpose:**

To develop a deep network architecture that would achieve fully automated radiologist-level segmentation of cancers at breast MRI.

**Materials and Methods:**

In this retrospective study, 38 229 examinations (composed of 64 063 individual breast scans from 14 475 patients) were performed in female patients (age range, 12–94 years; mean age, 52 years ± 10 [standard deviation]) who presented between 2002 and 2014 at a single clinical site. A total of 2555 breast cancers were selected that had been segmented on two-dimensional (2D) images by radiologists, as well as 60 108 benign breasts that served as examples of noncancerous tissue; all these were used for model training. For testing, an additional 250 breast cancers were segmented independently on 2D images by four radiologists. Authors selected among several three-dimensional (3D) deep convolutional neural network architectures, input modalities, and harmonization methods. The outcome measure was the Dice score for 2D segmentation, which was compared between the network and radiologists by using the Wilcoxon signed rank test and the two one-sided test procedure.

**Results:**

The highest-performing network on the training set was a 3D U-Net with dynamic contrast-enhanced MRI as input and with intensity normalized for each examination. In the test set, the median Dice score of this network was 0.77 (interquartile range, 0.26). The performance of the network was equivalent to that of the radiologists (two one-sided test procedures with radiologist performance of 0.69–0.84 as equivalence bounds, *P* < .001 for both; *n* = 250).

**Conclusion:**

When trained on a sufficiently large dataset, the developed 3D U-Net performed as well as fellowship-trained radiologists in detailed 2D segmentation of breast cancers at routine clinical MRI.

**Keywords:** MRI, Breast, Segmentation, Supervised Learning, Convolutional Neural Network (CNN), Deep Learning Algorithms, Machine Learning Algorithms

Published under a CC BY 4.0 license.

*Supplemental material is available for this article.*

## Graphical abstract

**Figure:**
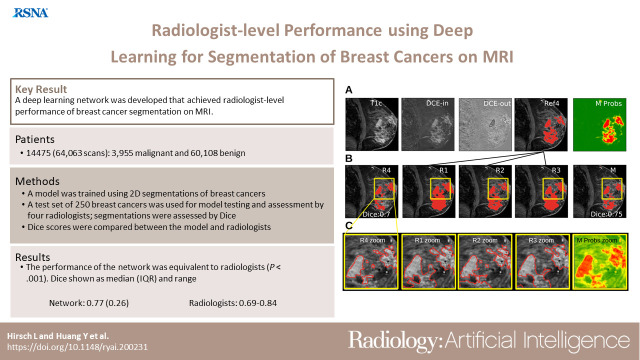

**Summary**

When trained on a sufficiently large dataset, a volumetric deep convolutional neural network achieved radiologist-level performance at segmenting breast cancers at MRI.

**Key Points**

■ Convolutional neural networks were developed to perform fully automated segmentation of breast cancer at MRI, leveraging a large dataset of more than 38 000 examinations for training.
■ The highest-performing network was a three-dimensional U-Net trained with routine clinical dynamic contrast-enhanced MRI; it achieved segmentation performance comparable to that of radiologists who evaluated an independent test set.
■ The code and pretrained network have been made freely available.

## Introduction

Segmentation of breast tumors provides image features such as shape, morphologic structure, texture, and enhancement dynamics that can improve diagnosis and prognosis in patients with breast cancer (1–3). To our knowledge, reliable automated tumor segmentation does not yet exist, and manual segmentation is labor intensive; this has precluded routine clinical evaluation of tumor volume despite mounting evidence that it is a good predictor of patient survival (2). Automatic segmentation with modern deep network techniques has the potential to meet this clinical need.

Deep learning methods have been applied in breast tumor segmentation (4,5) and diagnosis (6–11) on mammograms; large datasets of up to 1 million images are available, which greatly boosts the performance of the machine learning systems (12,13). Unlike MRI, however, mammography cannot depict the exact three-dimensional (3D) location and volumetric extent of a lesion. Breast MRI has a higher diagnostic accuracy than mammography (14–16) and outperforms mammography in detection of residual tumors after neoadjuvant therapy (17). Additionally, background parenchymal enhancement measured at MRI with dynamic contrast enhancement is predictive of cancer risk (18). Several studies have automated tumor segmentation in breast MRI by using modern deep networks such as U-Nets or DeepMedic (19–25), focusing mostly on malignant tumors. With reliable, fully automated segmentation, the overall clinical workflow could be improved, and such segmentation could help radiologists in tumor detection and diagnosis.

Fast automated segmentation may help identify important prognostic and predictive biomarkers. Unfortunately, the available MRI datasets to train segmentation algorithms are comparatively small, with 50–250 MRI examinations (19–25), which limits the potential of modern deep networks. Some studies have been limited to semiautomated segmentation (26), and performance differs across datasets, making comparison with radiologist performance difficult. In a study (20) in which a formal comparison was conducted on the same dataset, radiologists outperformed the networks.

We hypothesized that human-level performance could be achieved if a sufficiently large dataset was used to train a modern deep convolutional neural network. The goal of this research was to develop a deep network architecture that achieved fully automated, radiologist-level segmentation of breast cancer at MRI.

## Materials and Methods

### Study Design

This retrospective study was approved by the institutional review board, and written informed consent was waived because of the retrospective nature of this study. All data handling complied with Health Insurance Portability and Accountability Act regulations. The breast MRI examinations we analyzed may overlap with examinations analyzed in previous publications involving authors from the radiology department at Memorial Sloan Kettering Cancer Center.

**Figure 1:**
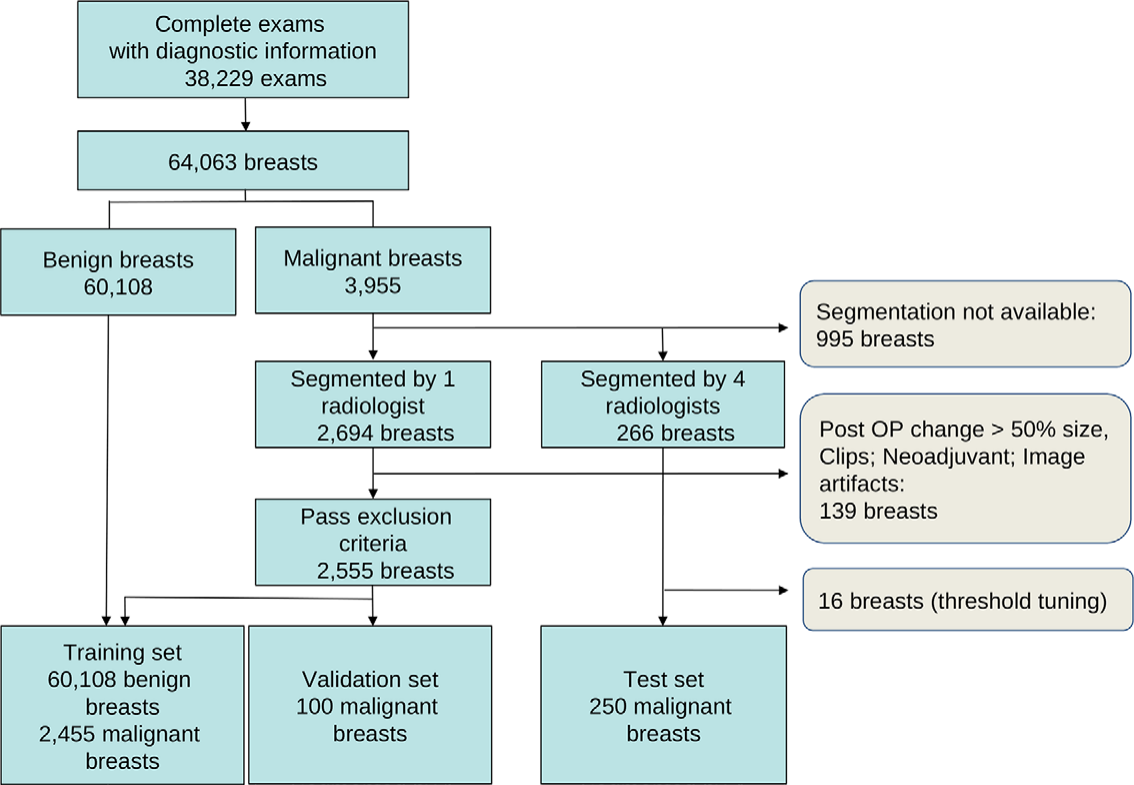
Number of examinations (exams) and breasts used in training and testing. See also Table E1 (supplement). Post OP = postoperative procedure.

The dataset was composed of 38 229 clinical breast MRI examinations performed from 2002 to 2014 for either high-risk screening (11 929 patients) or diagnostic purposes (2546 patients). The age range of the patient population was 12–94 years (mean age, 52 years ± 10 [standard deviation]). Unilateral and bilateral examinations were included, totaling 64 063 breasts (Fig 1). Of these, 3955 breasts had biopsy-confirmed malignant histopathologic findings (hereafter, referred to as malignant breasts), and 60 108 had benign histopathologic findings or showed 2 years of imaging stability (Breast Imaging Reporting and Data System category 1, 2, or 3) and/or no clinical evidence of disease (hereafter, referred to as benign breasts). The types of tumors included in the study are listed in Table E2 (supplement). Exclusion criteria are described in Appendix E1 (supplement).

The data were randomly partitioned into training, validation, and test sets (Fig 1, Table E1 [supplement]), ensuring that each breast was included in only one of the three sets. For the purpose of training the segmentation network, voxels were labeled as positive for tumor or negative for tumor by using two-dimensional (2D) segmentations performed by fellowship-trained radiologists in malignant breasts. Training of the network also included voxels of noncancerous tissue from a central MRI section of benign breasts, which served as additional control examples negative for tumor. Therefore, the network was trained to distinguish cancerous from noncancerous tissue. By using the training data, we first selected the highest-performing network architecture, established the value of the available imaging sequences, and selected an effective harmonization procedure.

We then compared the segmentations produced by the final network to those produced by fellowship-trained radiologists in a separate test set of 250 malignant breasts that were each segmented independently by four radiologists in 2D (see below). Benign breasts were not included in this analysis. The purpose of our analysis was to determine if the performance of the network was equivalent to the performance of fellowship-trained radiologists at segmenting histopathologically confirmed cancers.

### Data Description and Harmonization

**Figure 2:**
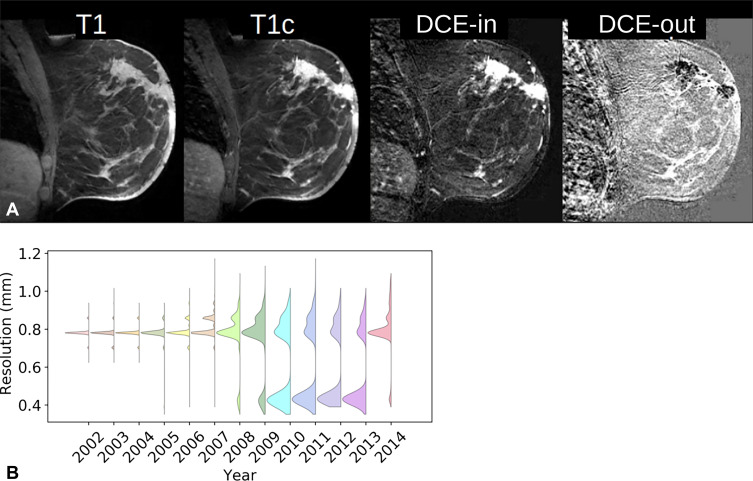
**(A)** Example of precontrast and first postcontrast fat-saturated images (T1 and T1c, respectively). Initial dynamic contrast enhancement (DCE) in this breast with malignant tumor is evident after subtracting the first T1-weighted contrast-enhanced image from the precontrast image (DCE-in). Subsequent washout (DCE-out) is evident in the subsequent drop in intensity, measured as slope over time. **(B)** Graph shows the range of in-plane resolutions of T1-weighted contrast-enhanced scans acquired between 2002 and 2014.

All breast MRI examinations were performed with a 1.5-T or 3.0-T scanner (Signa Excite, Genesis Signa, Discovery, Signa HDxt, and Optima MR450w; GE Healthcare). Examinations were performed on the sagittal plane (Fig 2A) at varying in-plane resolutions (Fig 2B), at a section thickness of 2–4 mm, and at varying repetition times and echo times. The sequences used from each MRI examination included fat-saturated T2- and T1-weighted images obtained before the administration of contrast material (hereafter, precontrast T1-weighted image) and a varied number (*n* = 3–8) of fat-saturated T1-weighted images obtained after the administration of contrast material (hereafter, postcontrast T1-weighted image). In-plane sagittal resolution was harmonized by upsampling relatively low-spatial-resolution images by a factor of two (Fig 2B). Image intensity data from different scanners were harmonized by dividing by the 95th percentile of precontrast T1 intensity. To summarize the dynamic contrast enhancement, we measured the volume transfer constant for the initial uptake by using the following equation: first postcontrast T1-weighted image – precontrast T1-weighted image (referred to as dynamic contrast enhancement in), and subsequent washout (linear slope of intensity divided by time in the postcontrast T1-weighted image images, referred to as dynamic contrast enhancement out) (Fig 2A). Data collection, preprocessing, and harmonization are described in Appendix E1 (supplement).

### Radiologist Segmentations

All segmentations were performed on 2D sections by fellowship-trained breast radiologists (R1–R10: I.D.N., A.G.V.B., R.L.G., C.R.S., E.J.S., M.H., N.O., E.S.K., D.A., and D.L., with 5, 8, 5, 5, 8, 12, 7, 13, 5, and 2 years of experience, respectively). At the start of this project, 2694 breasts had been segmented by individual radiologists (R1–R10) by outlining the malignant tissue in a single section. These segmentations were subsequently reviewed by R1–R5 to ensure they met the inclusion criteria, resulting in 2555 segmentations used for training and validation (Fig 1, Table E1 [supplement]; 2455 segmentations used for training and 100 for validation). An additional 266 breast cancers were independently segmented by all four radiologists (R1–R4); of these cancers, 16 were used for threshold tuning and 250 for testing. The common 2D section to be segmented, containing the largest area of the index cancer, was selected by R5. Radiologists performed segmentations on the postcontrast T1-weighted image, with the T1-weighted and fat-saturated T2-weighted images available for reference. For the test data, we also provided the dynamic contrast enhancement–in value, which quantifies initial uptake. See Appendix E1 (supplement) for details.

### Convolutional Neural Networks

**Figure 3:**
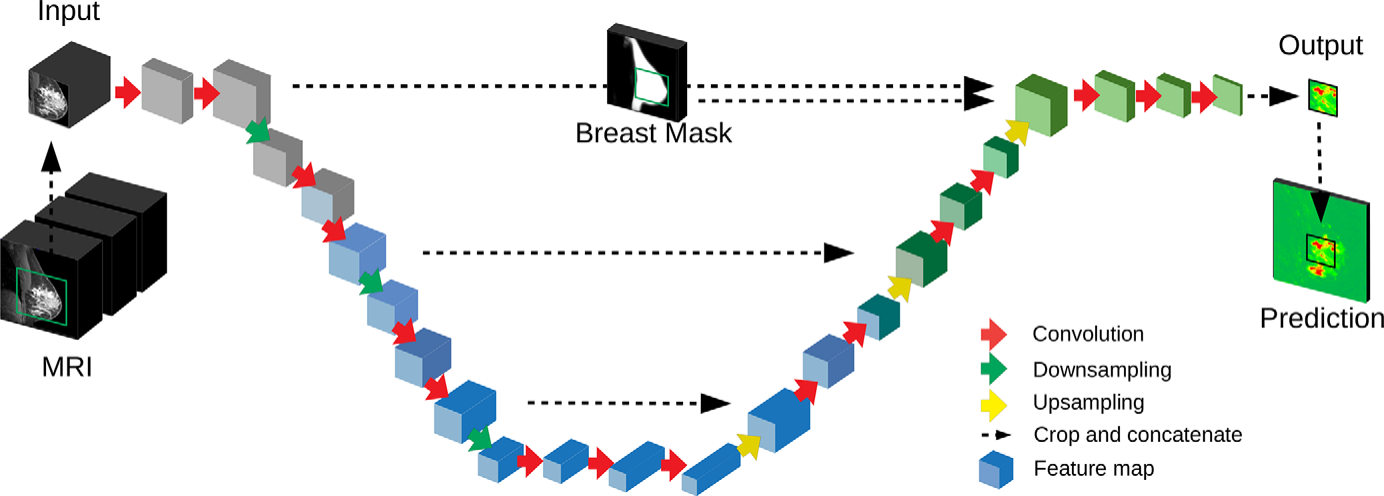
Deep convolutional neural network used for segmentation. A three-dimensional (3D) U-Net with a total of 16 convolutional layers (red arrows) resulting in 3D feature maps (blue blocks). The input MRI includes several modalities (Fig 2A). The network output is a prediction for a two-dimensional sagittal section, with probabilities for cancer for each voxel (green and red map). The full volume is processed in nonoverlapping image patches (green square on input MRI). A breast mask provides a spatial prior as input to the U-Net.

We used networks based on the DeepMedic network (27) and a 3D U-Net (20), which have been used extensively for medical segmentation, including breast segmentation (19–24). The architecture of the 3D U-Net is described in Figure 3 and that of DeepMedic in Figure E2 (supplement). Following previous studies, the traditional space-invariant implementation has been augmented by adding a spatial prior as input to the final classification (28). For the U-Net, the spatial prior was a breast mask, as in previous studies (20). This breast mask was computed by using a separate U-Net operating on the entire image at lower spatial resolution. This network was trained on a smaller number of manually segmented breast sections (*n* = 100; performed by L.H.). To avoid blocking artifacts that are often observed in U-Nets (29), we carefully redesigned the conventional downsampling and upsampling steps. The 3D U-Net had approximately 3 million parameters. Details of the architectures, sampling, and training of the network parameters are described in Figures E2 and E3 (supplement).

### Primary Outcome Measure

**Figure 4:**
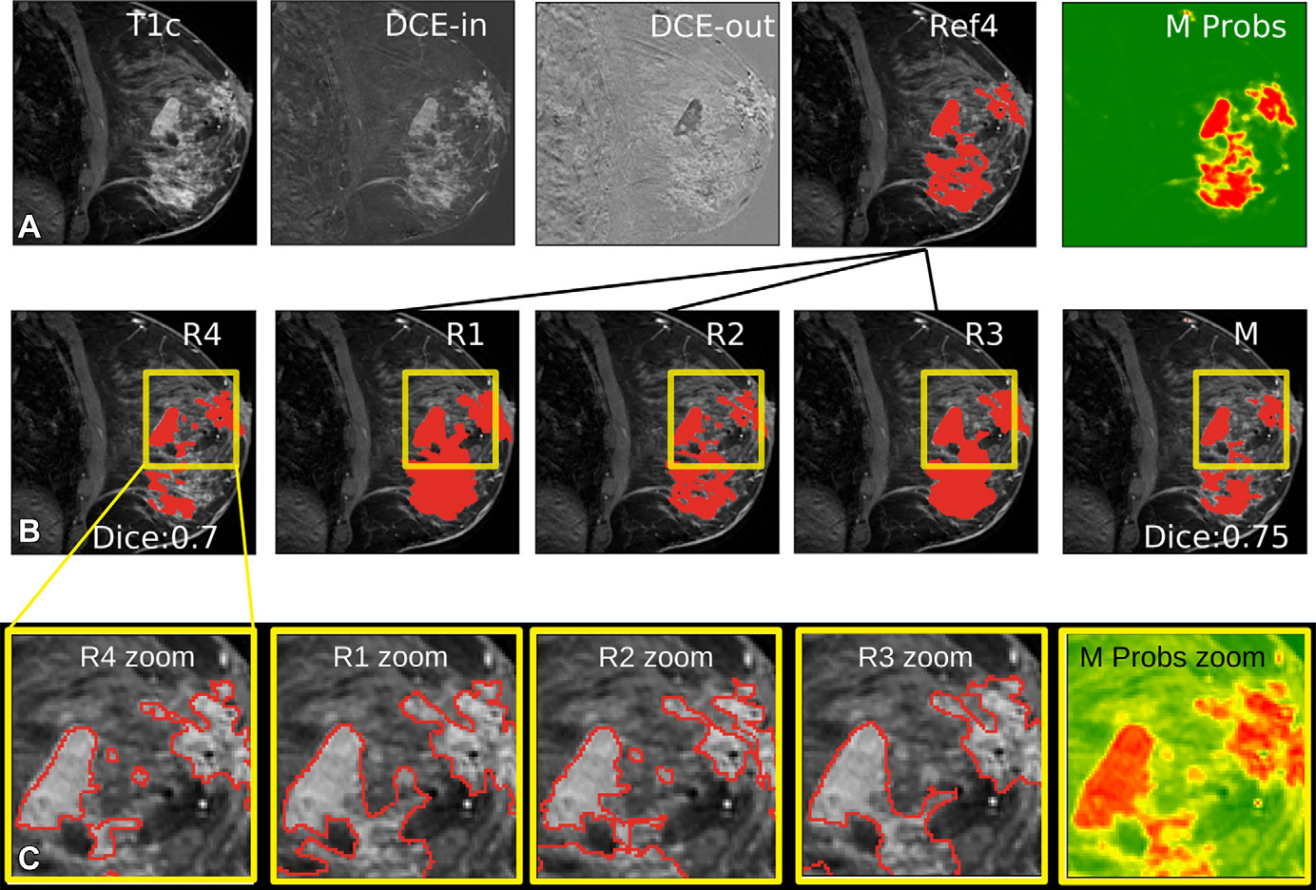
Manual and automated segmentations of breast cancer. **(A)** Inputs to the model consisting of the first postcontrast image (T1c), postcontrast minus precontrast image (T1) (DCE-in), and washout (DCE-out), with an independent reference for radiologist 4 (R4) made from the intersection of radiologists 1–3 (R1–R3 [Ref4]) and the network output (M Probs) indicating probability that a voxel is cancer (green = low; red = high). **(B)** Example segmentation from all four radiologists (R1–R4) for a given section, and the model segmentation created by thresholding probabilities (M). Dice scores for R4 and M were computed using Ref4 as the target. **(C)** Zooming in on the areas outlined in yellow in **B**, showing the boundaries of segmentations for the machine as well as human-generated segmentations as drawn on the screen by R1–R4.

For each voxel, the network estimates the probability of the voxel being part of the cancerous tissue (Fig 4A). A binary segmentation was obtained by thresholding this probability at a fraction of the maximum in the selected section (Fig 4B) and dismissing disconnected areas that did not reach the maximum. The primary outcome measure was the Dice score (30) for the cancer evaluated on the 2D section, with consensus segmentation as the reference (Fig 4A; Appendix E1 [supplement]). A Dice score of 1.0 corresponds to perfect overlap, and a score of 0.0 indicates no overlap. To determine the sample size required for the comparison between the network and radiologists, we assumed a mean Dice score of 0.75 as the lower bound for the radiologist and of 0.80 as the lower bound for the network. Power analysis for a paired *t* test was performed with logit-transformed Dice scores to approximate normality (31). Use of an estimated standard deviation of 1.5 across examinations (based on the network performance in the validation set) resulted in Cohen *d* of 0.1918. With this effect size, a power of 85% at a significance of 5% required 246 scans. We selected a sample size of 250 scans for the test set.

### Statistical Analysis

All pairwise performance comparisons among different network architectures and between the network and the radiologists were performed by using the Wilcoxon signed rank test on the Dice score. This analysis accounted for the deviation from normality observed for the Dice scores (Shapiro-Wilk test, *W* = 0.61, *P* < .001 for difference in mean Dice score; logit-transformed Dice scores, *W* = 0.92, *P* < .001). We tested for equivalence in the Dice score of human and machine by using the two one-sided test procedure (32). In this procedure, performance is compared with a lower and upper equivalence bound, for which we selected the lowest- and highest-performing radiologists. The Wilcoxon signed rank test was used instead of the conventional *t* test of the two one-sided test procedure because Dice scores were not normally distributed. Effect size of the Wilcoxon test is reported as described by Vargha and Delaney (33). Values are medians ± interquartile ranges. All statistical analyses were performed and implemented by using the software Python 2.7, package scipy.stats (version 1.2.3; Python Software Foundation).

### Model Availability

To facilitate such studies, we have made all code and the pretrained network freely available in Github (*https://github.com/lkshrsch/Segmentation_breast_cancer_MRI/*).

## Results

### Model Development, Initial Assessment, and Model Selection

To select the preferred network architecture, input modalities, and harmonization method, we trained various architectures (Appendix E1 [supplement]). We evaluated performance on a validation set of 100 scans with malignant results. On the basis of these results (Fig E4 [supplement]), we selected a volumetric implementation of a 3D U-Net (Fig 3), which takes as inputs the first postcontrast T1-weighted image, the initial uptake (postcontrast T1-weighted image − precontrast T1-weighted image), and the slope of the subsequent postcontrast T1-weighted images (Fig 2A). These sagittal images were aligned with deformable coregistration (34) covering the volume of a single breast. Intensity was harmonized by scaling each examination separately with a maximum of the precontrast T1-weighted image. When training this network with datasets of different sizes, our hypothesis that larger datasets significantly improve Dice score performance was supported, with median values of 0.63 (240 scans with malignant results and 240 scans with benign results), 0.69 (2400 scans with malignant results and 2400 scans with benign results), and 0.73 (2455 scans with malignant results and 60 108 scans with benign results), all evaluated on a separate validation set of 100 malignant breasts (Fig E5 [supplement]).

### Model Testing

**Figure 5:**
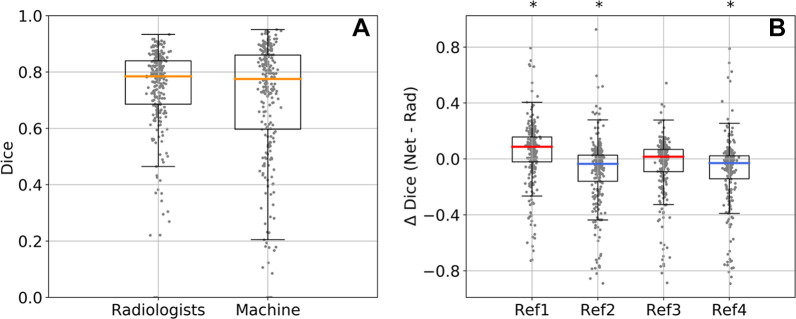
Network (net) and radiologist (rad) performance on the test set of 250 malignant cases. **(A)** Distribution of Dice scores in 250 test cases averaged across four reference segmentations. **(B)** Difference in Dice score between the network and each radiologist (Δ Dice) for each of the four reference (ref) segmentations (ref1, ref2, ref3, and ref4). The median Dice value was higher for the network for ref1 and ref3 (red median Δ Dice) and higher for the radiologist for ref2 and ref4 (blue median Δ Dice). Box plots show median (orange, red, or blue lines), quartiles (box), and 1.5 interquartile range (whiskers). *P < .001 (Wilcoxon signed rank test).

Performance of this final design was tested on an independent test set of 250 malignant cases. Although the network produced 3D segmentations, evaluation was limited to 2D segmentations, and cancers were segmented independently by four breast radiologists (R1–R4) on a single 2D section per breast (Fig 4). Segmentations differed across radiologists in the areas selected and on detailed boundaries (Fig 4C, Fig E9 [supplement]). An independent reference segmentation was obtained for each radiologist by using segmentations from the three remaining radiologists; for example, reference segmentation 1 is the intersection of the segmentations of R2–R4 and is used to evaluate R1 (Fig 4A). The threshold for converting continuous probabilities at the output of the network into binary segmentations was estimated by using a separate set of reference segmentations (16 not included in the test set; Fig 1, Fig E6A [supplement]). The resulting Dice score (averaged over the four references) had a 5th–95th percentile range of 0.43–0.90 for the radiologists and 0.21–0.92 for the network (Fig 5A). These median Dice scores did not differ significantly between the network and the radiologists (median, 0.77 ± 0.26 and 0.79 ± 0.15, respectively; effect size, 0.51; *P* = .72 [*n* = 250]). The median Dice scores for the network were 0.76 ± 0.26, 0.76 ± 0.26, 0.77 ± 0.28, and 0.76 ± 0.28, and for the radiologists they were 0.69 ± 0.2, 0.84 ± 0.14, 0.78 ± 0.13, and 0.84 ± 0.13, with one value for each of the four reference segmentations, indicating that the model may have had higher Dice scores than some radiologists but not others (Fig 5B). A similar result was obtained with repeated measures of analysis of variance (Appendix E1 [supplement]).

### Equivalence Testing

To test for equivalence, we performed a two one-sided test procedure (32) with radiologist performance as the lower and upper bounds for equivalence (R1 and R4, respectively [Fig 5B]). The Dice score of the network was higher than that of R1 (effect size, 0.37; *P* < .001; *n* = 250) and lower than that of R4 (effect size, 0.62; *P* < .001; *n* = 250). In total, the mean performance of the network and that of the radiologists were indistinguishable, and the median performance of the network was equivalent to that of the radiologists.

### Segmentation Comparison between the Network and Radiologists

**Figure 6:**
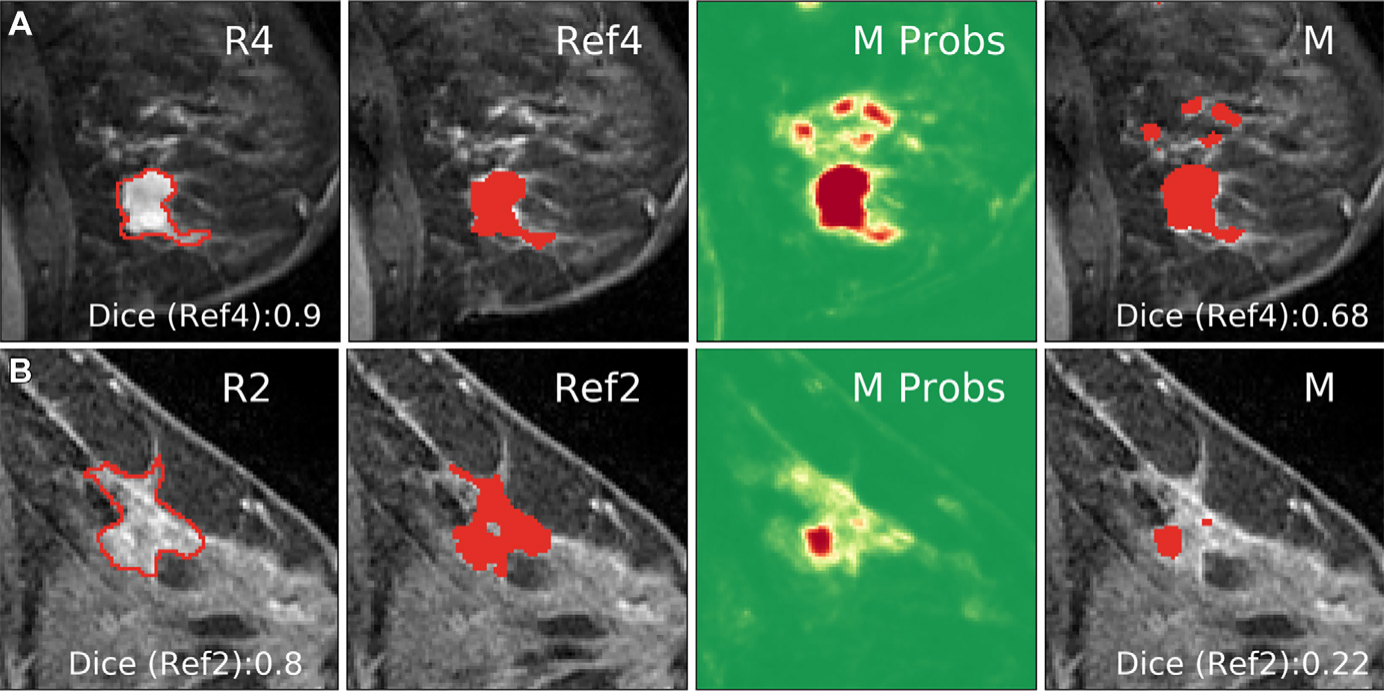
Examples of cases in which the network deviated from the segmentation of the reference radiologist (ref). **(A)** The network captured additional areas not selected by radiologist 4 (R4). Dice score shown for Ref4 (intersection of R1–R3). **(B)** The network output (M Probs) captured the correct area, but low probability values yielded a smaller region compared with the consensus segmentation (Ref2) after thresholding.

For several examinations the network had a higher performance than the average of the four radiologists (ΔDice > 0 [Fig 5B]; see Fig 4 and E9 [supplement] for examples). In several instances, however, the network had a lower performance than the radiologists (ΔDice < 0 [Fig 5B]; see Fig 6 for examples). The network deviated from the reference in the areas that it selected (Fig 6A) or the exact boundary of the cancer (Fig 6B). Network performance differed among tumor types (Fig E7A [supplement]) and was somewhat lower in the presence of prominent background parenchymal enhancement (Fig E7B [supplement]) and smaller cancer (Fig E7C [supplement]). Generally, images that had high Dice scores for the network also had high Dice scores for the radiologists, regardless of size or background parenchymal enhancement (Fig E8 [supplement]).

Similar results were obtained when the union or the majority vote of three radiologists as consensus reference was used (Fig E6 [supplement]). R2 and R4 had a higher performance than the network with the intersection as consensus reference (Fig 5B), whereas R1 and R3 had a higher performance than the network when compared with the union as consensus (Fig E6B [supplement]). This suggests that R2 and R4 opted for more specific tumor segmentation, whereas R1 and R3 provided more sensitive segmentations (Fig E9 [supplement]). As a reference, we also evaluated a conventional image segmentation method (fuzzy *c*-means [35]) and found poor performance on these data, which attested to the difficulty of the task (Dice score of 0.11, Fig E10 [supplement], and 0.65 when restricted to a limited region of interest, as in Chen et al [26], in which case the network Dice score was 0.82).

## Discussion

We demonstrated radiologist-level performance in fully automated segmentation of breast cancer at MRI by using routine precontrast and postcontrast T1-weighted images. The architecture of deep convolutional neural networks was optimized by using a large training dataset of more than 38 000 examinations, which consisted of 2555 malignant and 60 108 benign breast scans. This dataset is substantially larger than those in previous studies involving deep networks, which have used only 50–250 MRI examinations (19–24).

Notably, the highest performance was obtained with a volumetric U-Net that was conceptually simpler than previous networks (20,21,23). Complex structures with fewer parameters may have been necessary to compensate for the smaller dataset sizes used in these earlier studies. For instance, Zhang et al (20) combined three different 2D U-Nets in a hierarchical manner; one network generated a breast mask, a second network produced segmentations of tumors, and a third network refined these segmentations. We initially used a similar approach, following the MultiPrior network (28) with a breast mask as a spatial prior, and a conditional random field for postprocessing. However, we found that a simpler 3D U-Net without these additional modules sufficed. The U-Net also had a higher performance than DeepMedic (27), which we believe is the result of better integration of information at multiple spatial scales. Chen et al (21) used 2D U-Nets with a long short-term memory network at the input to process the contrast dynamic. Instead, we summarized the dynamic contrast enhancement on two images, capturing the initial contrast agent uptake and subsequent washout. This method allowed us to harmonize the differing sampling intervals and the number of postcontrast images and allowed the network to potentially capture a metric akin to the signal enhancement ratio, which has been proposed as a threshold criterion for dynamic contrast enhancement at breast MRI (36). El Adoui et al (23) used a Seg-Net that communicated only location information through residual connections, which reduced the number of parameters compared with a U-Net and therefore may have required fewer training images. Other previously developed network methods coped with smaller training sets by using preselected features (19) or an unsupervised clustering algorithm (24), or by leveraging shape priors (22).

Our final U-Net implementation differs from previous approaches in two important ways. First, we used a full 3D network instead of a conventional 2D network that processed individual sections independently (12,21,23–25). Whereas this increased the number of network parameters, it also captured volumetric features missed at 2D processing. Our implementation also avoided sampling artifacts encountered in conventional implementations of U-Nets (29). Although we did this with an apparent increase in complexity, our approach obviated the need for less carefully designed architectures to unlearn sampling errors.

Previous efforts to apply machine learning to breast cancer segmentation reported Dice scores of 0.60–0.77 (19–25); however, the performance of these models has, to our knowledge, not been compared with that of radiologists. In a direct comparison, Zhang et al (20) reported a Dice score of 0.72 ± 0.28 for the network and 0.78 ± 0.30 for the radiologists. Dice scores of 0.7 are considered to be good agreement (31). In our study, automated segmentations matched the detailed segmentations of a radiologist, with a Dice score of 0.76–0.77. Radiologist performance in this study was in the range of 0.69–0.84, which is comparable to previous reports.

In our study, equivalence of network performance was demonstrated on a fixed test set, which is important given the variability observed across studies by using different datasets (19–25). The test set in our study included difficult cases with small cancers (<1 cm), multicentric cancers, and patients with breast implants, all of whom have often been excluded from previous studies. Note that network performance was higher on the test set than on the validation set (0.77 vs 0.73), which may have resulted from more careful manual segmentations when radiologists were evaluated. Regardless, the performance of the radiologists, as well as the limited performance of conventional semiautomatic image segmentation methods, point to the difficulty of segmenting an entire 2D section in detail on these diverse images.

Our study had limitations. The developed network classified each voxel in the image and therefore, in principle, provided volumetric segmentation. The main clinical value of this network would be to facilitate volumetric assessment, which is not broadly used despite its benefit (2,3). However, we evaluated automated segmentations only on 2D sections because we expected higher radiologist performance compared with highly labor-intensive 3D manual segmentation. Another limitation was that we used sagittal images; assessment in that plane was standard practice at our institution until 2014, and it characterized most of the historical data. Breast MRI protocols are often performed on the axial plane and, with continued improvements in technology, with higher temporal and spatial resolution. Future studies could focus on volumetric evaluation of segmentations. Additionally, for high-spatial-resolution multiplanar breast MRI, one might expect to achieve higher segmentation performance for both the network and radiologists. Additionally, this study was retrospective and limited to a single institution. The dataset was heterogeneous, however; it was collected during 12 years from different scanner types by using different magnet strengths (1.5 T and 3.0 T) and with different breast coils, which resulted in variable spatial and temporal resolutions. All these factors together added to the difficulty and clinical realism of this study. Finally, the power analysis in this study assumed normality of logit-transformed Dice scores. The resulting Dice scores did not follow a normal distribution, however; the study may therefore have been underpowered.

In conclusion, when trained on a sufficiently large dataset, a 3D U-Net segmented breast cancers with performance comparable to that of fellowship-trained radiologists. The network produced detailed 3D segmentations in routine clinical MRI. The code and pretrained network were made freely available.

## References

[1] Bhooshan N, Giger ML, Jansen SA, Li H, Lan L, Newstead GM. Cancerous breast lesions on dynamic contrast-enhanced MR images: computerized characterization for image-based prognostic markers. Radiology 2010;254(3):680–690.20123903 10.1148/radiol.09090838 PMC2826695

[2] Hylton NM, Gatsonis CA, Rosen MA, et al. Neoadjuvant Chemotherapy for Breast Cancer: Functional Tumor Volume by MR Imaging Predicts Recurrence-free Survival-Results from the ACRIN 6657/CALGB 150007 I-SPY 1 TRIAL. Radiology 2016;279(1):44–55.26624971 10.1148/radiol.2015150013 PMC4819899

[3] Drukker K, Li H, Antropova N, Edwards A, Papaioannou J, Giger ML. Most-enhancing tumor volume by MRI radiomics predicts recurrence-free survival “early on” in neoadjuvant treatment of breast cancer. Cancer Imaging 2018;18(1):12.29653585 10.1186/s40644-018-0145-9 PMC5899353

[4] de Moor T, Rodriguez-Ruiz A, Mérida AG, Mann R, Teuwen J. Automated soft tissue lesion detection and segmentation in digital mammography using a u-net deep learning network. arXiv 1802.06865 [preprint] http://arxiv.org/abs/1802.06865. Posted February 19, 2018. Accessed April 29, 2020.

[5] Kallenberg M, Petersen K, Nielsen M, et al. Unsupervised Deep Learning Applied to Breast Density Segmentation and Mammographic Risk Scoring. IEEE Trans Med Imaging 2016;35(5):1322–1331.26915120 10.1109/TMI.2016.2532122

[6] Dhungel N, Carneiro G, Bradley A. The Automated Learning of Deep Features for Breast Mass Classification from Mammograms. In: Ourselin S, Joskowicz L, Sabuncu M, Unal G, Wells W, eds. Medical Image Computing and Computer-Assisted Intervention – MICCAI 2016. MICCAI 2016. Lecture Notes in Computer Science, vol 9901. Cham, Switzerland: Springer, 2016; 106–114.

[7] Dhungel N, Carneiro G, Bradley AP. Automated Mass Detection in Mammograms Using Cascaded Deep Learning and Random Forests. In: 2015 International Conference on Digital Image Computing: Techniques and Applications (DICTA), Adelaide, Australia, November 23–25, 2015. Piscataway, NJ: IEEE, 2015, 1–8.

[8] Kooi T, Litjens G, van Ginneken B, et al. Large scale deep learning for computer aided detection of mammographic lesions. Med Image Anal 2017;35:303–312.27497072 10.1016/j.media.2016.07.007

[9] Becker AS, Marcon M, Ghafoor S, Wurnig MC, Frauenfelder T, Boss A. Deep Learning in Mammography: Diagnostic Accuracy of a Multipurpose Image Analysis Software in the Detection of Breast Cancer. Invest Radiol 2017;52(7):434–440.28212138 10.1097/RLI.0000000000000358

[10] Zhu W, Lou Q, Vang YS, Xie X. Deep Multi-instance Networks with Sparse Label Assignment for Whole Mammogram Classification. arXiv 1612.05968 [preprint] http://arxiv.org/abs/1612.05968. Posted December 18, 2016 Accessed April 29, 2020.

[11] Ribli D, Horváth A, Unger Z, Pollner P, Csabai I. Detecting and classifying lesions in mammograms with Deep Learning. Sci Rep 2018;8(1):4165.29545529 10.1038/s41598-018-22437-z PMC5854668

[12] Wu N, Phang J, Park J, et al. Deep Neural Networks Improve Radiologists’ Performance in Breast Cancer Screening. IEEE Trans Med Imaging 2020;39(4):1184–1194.31603772 10.1109/TMI.2019.2945514 PMC7427471

[13] McKinney SM, Sieniek M, Godbole V, et al. International evaluation of an AI system for breast cancer screening. Nature 2020;577(7788):89–94.[Published correction appears in Nature 2020;586(7829):E19.] 31894144 10.1038/s41586-019-1799-6

[14] Warner E, Plewes DB, Hill KA, et al. Surveillance of BRCA1 and BRCA2 mutation carriers with magnetic resonance imaging, ultrasound, mammography, and clinical breast examination. JAMA 2004;292(11):1317–1325.15367553 10.1001/jama.292.11.1317

[15] Lehman CD, Isaacs C, Schnall MD, et al. Cancer yield of mammography, MR, and US in high-risk women: prospective multi-institution breast cancer screening study. Radiology 2007;244(2):381–388.17641362 10.1148/radiol.2442060461

[16] Chiarelli AM, Prummel MV, Muradali D, et al. Effectiveness of screening with annual magnetic resonance imaging and mammography: results of the initial screen from the ontario high risk breast screening program. J Clin Oncol 2014;32(21):2224–2230.24934793 10.1200/JCO.2013.52.8331

[17] Marinovich ML, Houssami N, Macaskill P, et al. Meta-analysis of magnetic resonance imaging in detecting residual breast cancer after neoadjuvant therapy. J Natl Cancer Inst 2013;105(5):321–333.23297042 10.1093/jnci/djs528

[18] Dontchos BN, Rahbar H, Partridge SC, et al. Are Qualitative Assessments of Background Parenchymal Enhancement, Amount of Fibroglandular Tissue on MR Images, and Mammographic Density Associated with Breast Cancer Risk? Radiology 2015;276(2):371–380.25965809 10.1148/radiol.2015142304 PMC4554209

[19] Wu H, Gallego-Ortiz C, Martel A. Deep Artiﬁcial Neural Network Approach to Automated Lesion Segmentation in Breast. In: Harz M, Mertzanidou T, Hipwell J, eds. Proceedings of the 3rd MICCAI Workshop on Breast Image Analysis, Munich, Germany, October 9, 2015. München, Germany: Fraunhofer Publica, 2015; 73–80.

[20] Zhang J, Saha A, Zhu Z, Mazurowski MA. Hierarchical Convolutional Neural Networks for Segmentation of Breast Tumors in MRI With Application to Radiogenomics. IEEE Trans Med Imaging 2019;38(2):435–447.30130181 10.1109/TMI.2018.2865671

[21] Chen M, Zheng H, Lu C, Tu E, Yang J, Kasabov N. A Spatio-Temporal Fully Convolutional Network for Breast Lesion Segmentation in DCE-MRI. In: Cheng L, Leung A, Ozawa S, eds. Neural Information Processing. ICONIP 2018. Lecture Notes in Computer Science, vol 11307. Cham, Switzerland: Springer, 2018; 358–368.

[22] Maicas G, Carneiro G, Bradley AP. Globally optimal breast mass segmentation from DCE-MRI using deep semantic segmentation as shape prior. In: 2017 IEEE 14th International Symposium on Biomedical Imaging (ISBI 2017), Melbourne, Australia, April 18–21, 2017. Piscataway, NJ: IEEE, 2017; 305–309.

[23] El Adoui M, Mahmoudi SA, Larhmam B, Benjelloun M. MRI Breast Tumor Segmentation Using Different Encoder and Decoder CNN Architectures. Computers 2019;8(3):52.

[24] Parekh VS, Macura KJ, Harvey SC, et al. Multiparametric deep learning tissue signatures for a radiological biomarker of breast cancer: Preliminary results. Med Phys 2020;47(1):75–88.31598978 10.1002/mp.13849 PMC7003775

[25] Spuhler KD, Ding J, Liu C, et al. Task-based assessment of a convolutional neural network for segmenting breast lesions for radiomic analysis. Magn Reson Med 2019;82(2):786–795.30957936 10.1002/mrm.27758 PMC6510591

[26] Chen W, Giger ML, Bick U. A fuzzy c-means (FCM)-based approach for computerized segmentation of breast lesions in dynamic contrast-enhanced MR images. Acad Radiol 2006;13(1):63–72.16399033 10.1016/j.acra.2005.08.035

[27] Kamnitsas K, Ledig C, Newcombe VFJ, et al. Efficient multi-scale 3D CNN with fully connected CRF for accurate brain lesion segmentation. Med Image Anal 2017;36(61):78.10.1016/j.media.2016.10.004 27865153

[28] Hirsch L, Huang Y, Parra LC. Segmentation of lesioned brain anatomy with deep volumetric neural networks and multiple spatial priors achieves human-level performance. arXiv 1905.10010 [preprint] http://arxiv.org/abs/1905.10010. Posted May 24, 2019. Accessed February 3, 2020.

[29] Aitken A, Ledig C, Theis L, Caballero J, Wang Z, Shi W. Checkerboard artifact free sub-pixel convolution: A note on sub-pixel convolution, resize convolution and convolution resize. arXiv 1707.02937 [preprint] http://arxiv.org/abs/1707.02937. Posted July 10, 2017. Accessed May 6, 2020.

[30] Dice LR. Measures of the Amount of Ecologic Association Between Species. Ecology 1945;26(3):297–302.

[31] Zou KH, Warfield SK, Bharatha A, et al. Statistical validation of image segmentation quality based on a spatial overlap index. Acad Radiol 2004;11(2):178–189.14974593 10.1016/S1076-6332(03)00671-8 PMC1415224

[32] Walker E, Nowacki AS. Understanding equivalence and noninferiority testing. J Gen Intern Med 2011;26(2):192–196.20857339 10.1007/s11606-010-1513-8 PMC3019319

[33] Vargha A, Delaney HD. A Critique and Improvement of the CL Common Language Effect Size Statistics of McGraw and Wong. J Educ Behav Stat 2000;25(2):101–132.

[34] Rueckert D, Sonoda LI, Hayes C, Hill DLG, Leach MO, Hawkes DJ. Nonrigid registration using free-form deformations: application to breast MR images. IEEE Trans Med Imaging 1999;18(8):712–721.10534053 10.1109/42.796284

[35] Bezdek JC, Ehrlich R, Full W. FCM: The fuzzy c-means clustering algorithm. Comput Geosci 1984;10(2-3):191–203.

[36] Li KL, Henry RG, Wilmes LJ, et al. Kinetic assessment of breast tumors using high spatial resolution signal enhancement ratio (SER) imaging. Magn Reson Med 2007;58(3):572–581.17685424 10.1002/mrm.21361 PMC4508009

